# Breast Cancer Downstaging Practices and Breast Health Messaging Preferences Among a Community Sample of Urban and Rural Ugandan Women

**DOI:** 10.1200/JGO.2015.001198

**Published:** 2016-05-11

**Authors:** John R. Scheel, Yamile Molina, Donald L. Patrick, Benjamin O. Anderson, Gertrude Nakigudde, Constance D. Lehman, Beti Thompson

**Affiliations:** **John R. Scheel**, **Donald L. Patrick**, and **Benjamin O. Anderson**, University of Washington; **John R. Scheel**, Seattle Cancer Care Alliance; **Benjamin O. Anderson** and **Beti Thompson**, Fred Hutchinson Cancer Research Center, Seattle, WA; **Yamile Molina**, University of Illinois at Chicago, Chicago, IL; **Gertrude Nakigudde**, Uganda Women’s Cancer Support Organization, Kampala, Uganda; and **Constance D. Lehman**, Massachusetts General Hospital, Boston, MA.

## Abstract

**Purpose:**

Among a community sample of Ugandan women, we provide information about breast cancer downstaging practices (breast self-examination, clinical breast examination [CBE]) and breast health messaging preferences across sociodemographic, health care access, and prior breast cancer exposure factors.

**Methods:**

Convenience-based sampling was conducted to recruit Ugandan women age 25 years and older to assess breast cancer downstaging practices as well as breast health messaging preferences to present early for a CBE in the theoretical scenario of self-detection of a palpable lump (breast health messaging preferences).

**Results:**

The 401 Ugandan women who participated in this survey were mostly poor with less than a primary school education. Of these women, 27% had engaged in breast self-examination, and 15% had undergone a CBE. Greater breast cancer downstaging practices were associated with an urban location, higher education, having a health center as a regular source of care, and receiving breast cancer education (*P* < .05). Women indicated a greater breast health messaging preference from their provider (66%). This preference was associated with a rural location, having a health center as a regular source of care, and receiving breast cancer education (*P* < .05).

**Conclusion:**

Most Ugandan women do not participate in breast cancer downstaging practices despite receipt of breast cancer education. However, such education increases downstaging practices and preference for messaging from their providers. Therefore, efforts to downstage breast cancer in Uganda should simultaneously raise awareness in providers and support improved education efforts in the community.

## INTRODUCTION

Breast cancer incidence in Uganda, like many other low- and middle-income countries (LMICs) in sub-Saharan Africa (SSA), has been increasing by a staggering 5.2% per year for the past 15 years.^1^ Unlike most SSA countries, Uganda offers cancer treatment, including surgery, radiation, and chemotherapy, at no cost through the Ugandan Cancer Institute (UCI) and a collaborative arrangement with the Ugandan Ministry of Health, the US National Cancer Institute, and the Fred Hutchinson Cancer Research Center.^2^ Nonetheless, late-stage presentation is a primary obstacle to improving breast cancer outcomes in Uganda, where > 77% of women are given a diagnosis of advanced-stage disease, including 26% with metastatic stage IV cancer at initial presentation.^3,4^ In a recent analysis of patients with breast cancer treated at UCI, 187 presented with stage III or IV disease and had a < 40% chance of surviving 5 years; by contrast, no deaths occurred at 5 years for the 22 patients who presented initially with stage I or II disease.^4^ Thus, an understanding of the systems-based factors that contribute to late-stage presentation and may promote breast cancer downstaging is important to improving outcomes in Uganda and potentially other SSA countries where breast cancer treatment can be available.

In LMICs where population-based screening is neither practical nor affordable, early breast cancer detection requires active participation by both the patients and the health care system. According to guidelines from the American Cancer Society,^5^ the National Comprehensive Cancer Network,^6,7^ and the Breast Health Global Initiative,^8^ the importance of prompt reporting of new breast symptoms to a health professional should be emphasized. This requires breast awareness, which means that a woman should be able to identify significant changes in her breasts and needs to know that the reporting of these self-detected abnormalities can improve breast cancer outcome. In parallel, these women need access to clinics that can perform diagnostic work-ups to distinguish benign findings from cancers promptly.^9^ Thus, the evaluation of practices that reflect breast awareness education and clinical diagnostic services is relevant to improving downstaging.

Neither the teaching of breast self-examination (BSE) nor the performance of clinical breast examination (CBE) has been demonstrated in a screening setting to independently reduce breast cancer mortality.^10,11^ Nonetheless, for countries like Uganda where women commonly first present with visually obvious breast masses or ulcerated tumors that have been present for many months or years, the assessment of BSE and CBE practices can serve as surrogate measures for essential factors that contribute to or defeat breast cancer downstaging. Work in rural Ghana has shown that breast cancer awareness education is associated with increased self-reported BSE and may link to improved breast cancer early detection and downstaging.^12^ Similarly, CBE is necessary for diagnostic evaluation of clinically detectable masses and thickenings and is a basic-level resource for breast diagnosis in health settings at all economic levels.^6,7,9^ Thus, the measurement of BSE and CBE practices is a relevant proxy for patient-determined (BSE) and clinic-determined (CBE) breast cancer downstaging practices in an LMIC where breast cancer screening is unavailable. Furthermore, understanding how breast health messaging preferences related to these factors vary across sociodemographic, health care access, and prior breast cancer exposure factors can inform future approaches and programs to better target downstaging among women who have access to treatment.

The objectives of the current study were to provide information about downstaging practices and breast health messaging preferences among Ugandan women 25 to 65 years old and to examine downstaging practices and breast health messaging preferences across sociodemographic, health care access, and prior breast cancer exposure factors.

## METHODS

### Procedure

This study was conducted between January and July 2014 in close collaboration with the Ugandan Women’s Cancer Support Organization (UWOCASO), a local group of breast cancer survivors. These Ugandan women are familiar with Ugandan culture and have experience with administering survey instruments and providing breast cancer education. After the development of the survey through multiple iterations and its translation from English (primary language of Uganda) to Luganda (common local language), we piloted the survey among a group of UWOCASO workers.

This study was exempt from Ugandan and US institutional review board review. Local guides and UWOCASO workers recruited women from the community for this study. We included asymptomatic women age 25 years and older with no personal history of breast cancer. Trained UWOCASO members interviewed eligible women individually in a semiprivate area. Participating women received a small financial incentive for their time and effort in accordance with local recommendations.

### Participants and Setting

We collected survey data from 401 participants as follows: 100 from the capital city and largest urban center Kampala (Kamwonkya [n = 50] and Namuwongo [n = 50] communities) and 301 from rural villages and communities in south central Uganda (Rakai District: Kakuuto County, Ssanje Community [n = 100] and Mannya Parish [n = 100]; Kooki County, Lwanda Parish [n = 100]). The population densities were 24,423 people/square mile for the urban centers and ranged from < 50 people/square mile (Kakuuto County) to 251 to 500 people/square mile (Kooki County) for the rural centers.^13^

### Measures

#### Sociodemographic, Health Care Access, and Prior Breast Cancer Exposure Factors

Sociodemographic information included geographic region (urban, rural), age (25 to 39, 40 to 49, and 50 to 74 years), ethnicity (Bantu, other), religion (Christian, other), intimate partner status (marital/living with partner, other), education (primary or less [≤ 7 years], more than primary [> 7 years]), and income (≤ 500,000 shillings, > 500,000 shillings). The annual income question was recategorized into a bivariate response because few participants reported income greater than the poverty level (approximately 1.5 million shillings/year).^14,15^ For health care access factors, women reported their regular source of care (health center, other [eg, self-care at home, traditional healer]) and their usual form of payment for care (self-pay, charity care, other [eg, private health insurance]). For prior breast cancer exposure, women self-reported whether they had a family history of breast cancer (no, yes) and whether they had ever received breast cancer education (no, yes).

#### Breast Cancer Downstaging Practices

Women reported their lifetime history of examining or observing their own breasts for palpable lumps (BSE: never, ever) and whether they had undergone a CBE by a health provider in the past year (no, yes).

#### Breast Health Messaging Preferences

Women indicated whose advice would most influence them in presenting early for a CBE in the theoretical scenario of self-detection of a palpable lump. Response categories were health providers, family/friends, and societal sources (advertisement by the government, television, or radio). Women were also asked where they would choose to go for a CBE (local health clinic, regional referral hospital, or other [eg, no preference, abroad]).

### Data Collection and Analysis

The Collaborative Data Services at the Fred Hutchinson Cancer Research Center entered the questionnaire data by using the DatStat Illume software package (Seattle, WA). We produced descriptive information about downstaging practices and breast health messaging preferences. We conducted χ^2^ tests to examine the relationships of downstaging practices and health care messaging preferences across sociodemographic, health care access, and prior breast cancer exposure factors. All statistical analyses were performed with SPSS software (IBM Corporation, Chicago, IL).

## RESULTS

Table 1 summarizes sociodemographic factors and health care factors. The median age for the 401 women surveyed was 38 years (25 to 74 years). Most were married or living with a partner (62%), had a primary education or less (66%), and had an annual household income below the 33% poverty line (50%). Most participants reported receipt of medical care from a health center (61%) and self-pay for their care (67%). For prior breast cancer exposure, 14% reported a family history of breast cancer, and 47% self-reported receipt of previous breast cancer education.

**Table 1:**
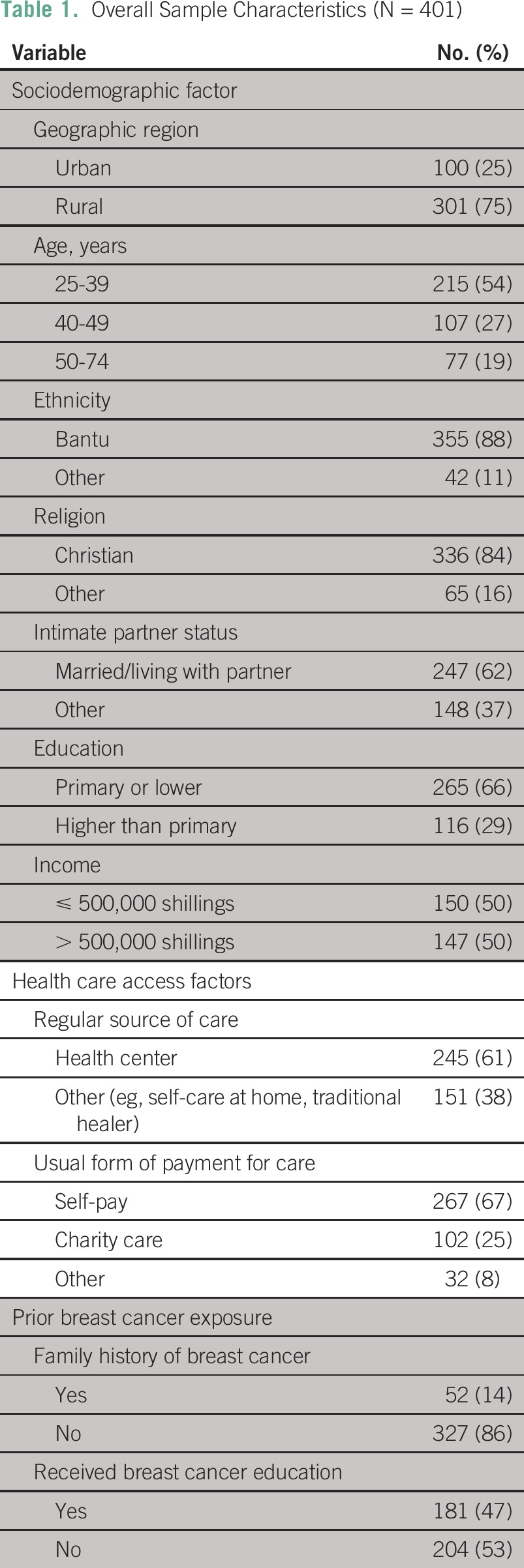
Overall Sample Characteristics (N = 401)

### Frequency of Downstaging Practices and Health Care Preferences

Table 2 depicts information about downstaging practices and breast health messaging preferences. Overall, the sample had low levels of downstaging practices: 27% had performed a BSE at least once in their lifetime and 15% had received a CBE in the past 12 months. Variability was found with regard to breast health messaging preferences: Women reported the greatest preference for breast health messaging by their health provider (66%) followed by friends/family (23%). Women preferred receipt of a CBE at a regional referral hospital (51%) to a local health clinic (12%).

**Table 2:**
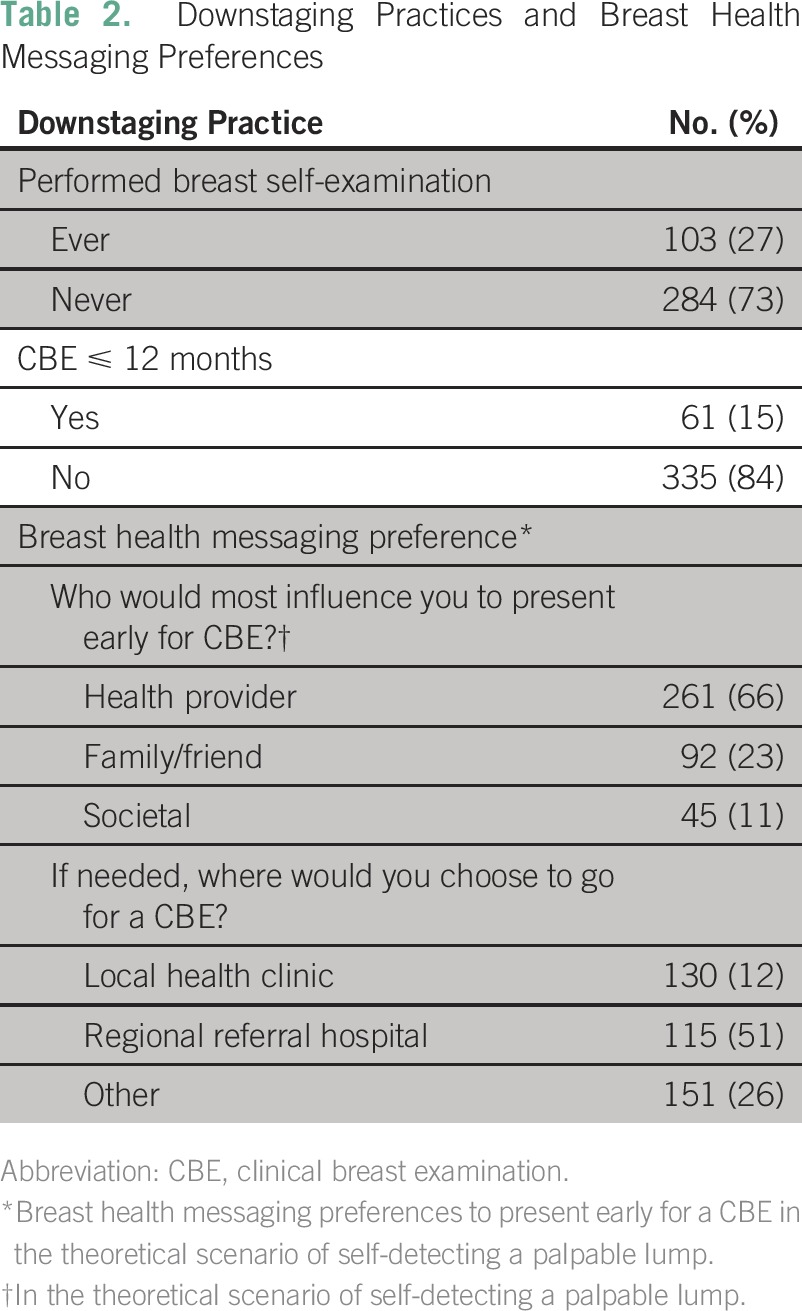
Downstaging Practices and Breast Health Messaging Preferences

### Variation of Downstaging Practices and Breast Health Messaging Preferences Across Sociodemographic, Health Care Access, and Prior Breast Cancer Exposure Factors

We next analyzed the distribution of downstaging practices across sociodemographic, health care access, and prior breast cancer exposure factors (Table 3). On the basis of geographic region, urban participants were significantly more likely to report on performing BSE (46% *v* 20%, *P* ≤ .001) and having a CBE in the past 12 months (34% *v* 9%, *P* ≤ .001) than their rural counterparts. Participants with more than a primary school education were more likely to perform BSE (39% *v* 21%, *P* ≤ .001). Women who received regular care at the health center also were more likely to receive a CBE in the past 12 months (20% *v* 9%, *P* = .004). Women who received previous breast cancer education showed significantly higher downstaging practices for both BSE (37% *v* 18%, *P* ≤ .001) and CBE (27% *v* 5%, *P* ≤ .001). No significant difference was found in downstaging practices related to age, marital status, income, usual pay, and family history.

**Table 3:**
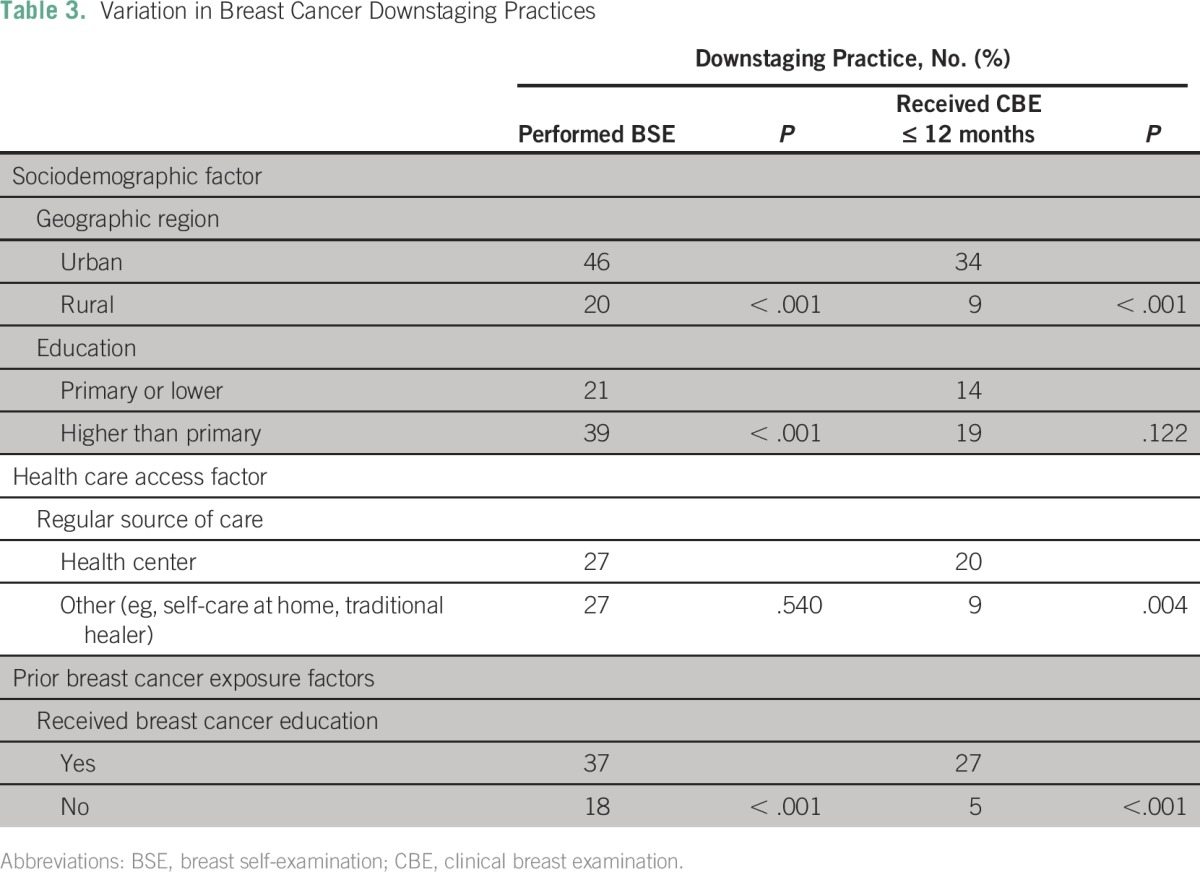
Variation in Breast Cancer Downstaging Practices

We also analyzed breast health messaging preference across sociodemographic, health care access, and prior breast cancer exposure factors (Table 4). Relative to urban counterparts, a greater proportion of rural women indicated that they preferred breast health messaging from their health provider (69% *v* 56%, *P* ≤ .001). Conversely, urban women showed a greater preference for breast health messages from societal factors after self-detection of a palpable lump (24% *v* 7%, *P* ≤ .001). With regard to health care access factors, women who reported health centers as the regular source of health care showed a greater preference for breast health messaging from their health providers (72% *v* 57%, *P* = .005). Women who self-paid for health care showed less preference for breast health messaging from their health providers compared with those who paid by other means (62% *v* 71% to 75%, *P* = .048) and a greater preference for breast health messaging from family/friends (28% *v* 9% to 16%, *P* = .048). Women who reported having received breast cancer education showed a greater preference for breast health messages from their health providers compared with those who reported no breast cancer education (70% *v* 58%, *P* = .021) and less preference from family/friends (18% *v* 30%, *P* = .021).

**Table 4:**
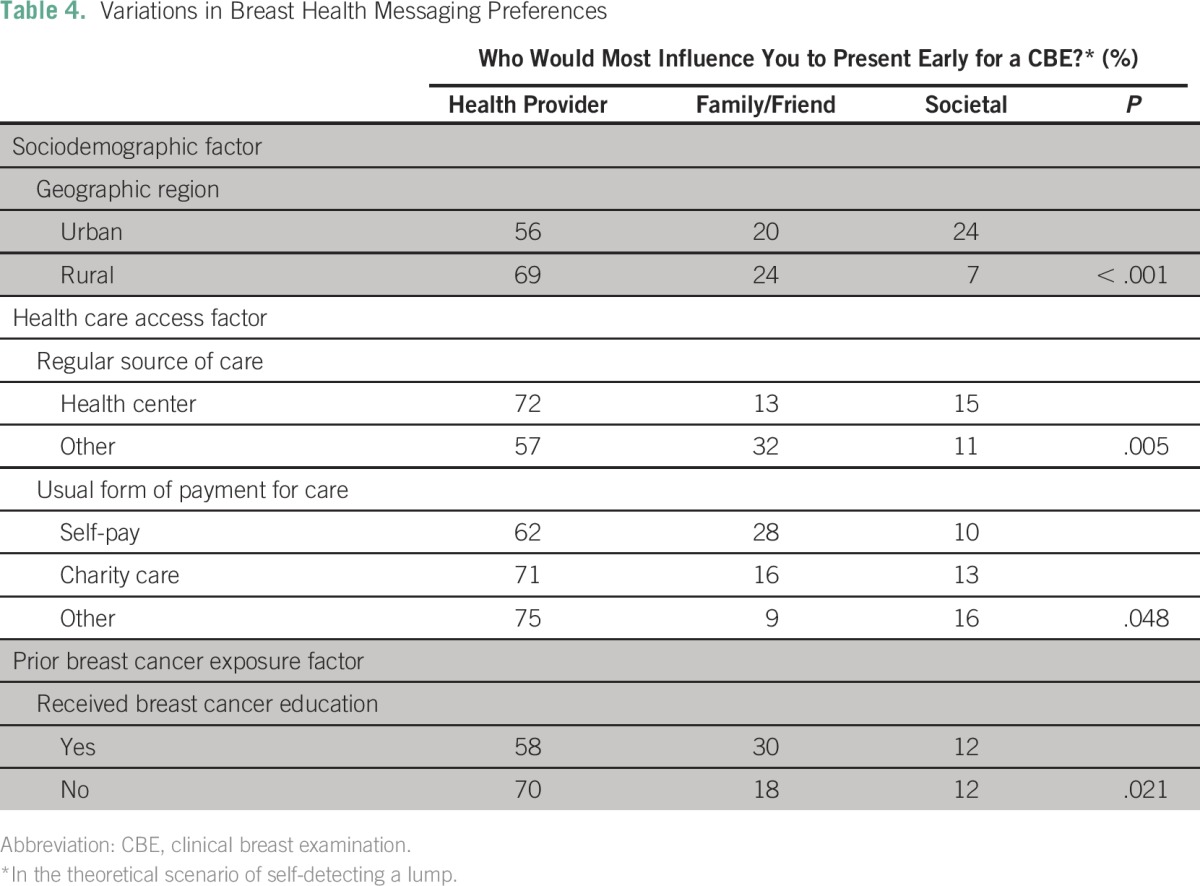
Variations in Breast Health Messaging Preferences

## DISCUSSION

Breast cancer is a growing problem in SSA and has the potential to overwhelm limited resources.^16,17^ The increasing incidence of breast cancer in LMICs places an enormous burden on individuals and their families in an already taxed health care system.^18,19^ For these reasons, the World Health Organization is leading efforts to reduce this avoidable late disease burden by 2025.^20^ Breast cancer treatment is available in Uganda, but these efforts are thwarted by late-stage presentation when 75% to 90% of such women receive a diagnosis of locally advanced (stage III) or metastatic (stage IV) disease.^3,4^ Even in the United States, where the latest treatment options are available, such late stages are associated with more costly and technically demanding treatment and poorer survival^9,21^; therefore, efforts should focus on detecting breast cancer at an earlier stage (downstaging).^22-24^

To inform interventions to improve outcomes, we surveyed Ugandan women to assess their baseline downstaging practices and breast health messaging preferences and to examine variations in these across sociodemographic, health care access, and prior breast cancer exposure factors. Uganda was chosen as the study site because the UCI offers breast cancer treatment at no cost to the patient. Unfortunately, improved access to treatment is not as effective against late-stage breast cancer as it is in early-stage breast cancer. Therefore, downstaging is a prerequisite to improve breast cancer outcomes in a limited resource setting.^23,25,26^

Before designing interventions, it is valuable to understand the populations’ baseline experiences with the downstaging practices and health messaging preferences (eg, providers, family/friends) that are likely to be effective.^27,28^ Two previous studies suggested that the majority of Ugandan women performed a BSE at least once and almost one half received a CBE in the past year.^29,30^ Both these studies were limited in their generalizability to the Ugandan population, with one focusing on breast cancer survivors and the other on patients who already accessed health care at the largest hospital in Uganda. An understanding of these downstaging practices and breast health messaging preferences in the general population would better inform interventions.

Before the present study, little was known about the variation in downstaging practices and breast health messaging preferences across sociodemographic, health care access, and prior breast cancer exposure factors in Uganda. Health care access factors in Uganda, such as where a woman receives her routine medical care and how medical care is usually paid for, are influenced by sociodemographic variables, including geographic region (rural *v* urban), education, and income.^14,31-33^ Although prior breast cancer exposure (positive family history and breast cancer education) could influence downstaging practices,^34-37^ this question had not been evaluated.

The present study confirms that downstaging practices and breast health messaging preferences vary by sociodemographic, health care access, and prior breast cancer exposure factors. We found that few Ugandan women participate in downstaging practices (BSE, 27%; CBE, 15%), despite what previous research has suggested (BSE, 60%; CBE, 40%).^29^ These differences may be related to our community-based sample compared with the sample used by Elsie et al^29^ that had already accessed the health care system. Within the present sample, we similarly noted that women who received their health care at a health clinic, and therefore accessed the health care system, were twice as likely to have a recent CBE. Although 54% reported having received prior breast cancer education and did not participate in downstaging practices, we simultaneously observed that women who received previous breast cancer education were twice as likely to have performed a BSE and more than five times more likely to have had a CBE than women who had not received breast cancer education. Such findings provide some support for the positive impacts of breast cancer education promoted by advocacy groups for improving practices in LMICs.^12,38^ These findings also emphasize the challenges facing downstaging efforts in LMICs and suggest that some barriers are not being addressed with current education efforts.

Our second objective was to identify sources of information most likely to be effective in communicating breast health information. We found in the present sample that 66% of women prefer breast health messaging from their health provider. These findings support previous studies that have shown the patient-provider relationship as the most important influence on health practices in Uganda.^39,40^ We also found that breast cancer education significantly increased preference for breast health messages from health providers. These findings suggest that education that targets providers may boost current efforts led by village health teams and nongovernmental organizations.

Although an improvement on prior survey studies, the convenience-based sampling used in the present study may limit its generalizability. Specifically, the urban and rural centers surveyed were mostly poor, and their residents had less than a primary school education. The middle class in SSA is growing, but still > 67% of Ugandans are poor or vulnerable to poverty and have little education.^15,31^ Thus, we believe that the present study population provides a reasonable estimate of most Ugandan women. We also acknowledge that other social factors and beliefs beyond those considered here may adversely affect the stage of diagnosis, such as the role of traditional healers in delaying presentation to the hospital. These factors go beyond the scope of the current analysis but warrant investigation, especially once standard early detection and diagnosis systems are established and functioning.

In summary, we conclude that knowledge of the variations in downstaging practices and breast health messaging preferences across various sociodemographic, health care access, and prior breast cancer exposure factors can help to inform future basic interventions. The findings suggest that by providing education to both health providers and women downstaging practices will improve, and this combined approach may be more effective in encouraging women to present early after self-detection of a lump.
